# Maternal and fetal risk factors for stillbirth in Northern Tanzania: A registry-based retrospective cohort study

**DOI:** 10.1371/journal.pone.0182250

**Published:** 2017-08-15

**Authors:** Francisca S. Chuwa, Amasha H. Mwanamsangu, Benjamin G. Brown, Sia E. Msuya, Elizabeth E. Senkoro, Oresta P. Mnali, Festo Mazuguni, Michael J. Mahande

**Affiliations:** 1 Department of Community Health, Institute of Public Health, Kilimanjaro Christian Medical College, Moshi, Tanzania; 2 Kilimanjaro Christian Medical University College, Moshi, Tanzania; 3 Department of Epidemiology & Biostatistics, Institute of Public Health, Kilimanjaro Christian Medical University College, Moshi, Tanzania; 4 Department of Global Health,Weill Cornell Medical College, New York, NY, United States of America; London School of Economics and Political Science, UNITED KINGDOM

## Abstract

**Background:**

Stillbirth is a major cause of perinatal mortality and occurs disproportionately in developing countries including Tanzania. However, there is scant information regarding the predictors of this condition in Tanzania. This study aimed to determine maternal and fetal risk factors for stilbirth in northen Tanzania.

**Methodology:**

A retrospective cohort study was performed using maternally-linked data from the Kilimanjaro Christian Medical Centre birth registry. A total of 47681 women who had singleton delivery at KCMC between 2000 and 2014 were analyzed. Women with multiple gestations were excluded. Descriptive statistics were summarized using proportions and frequency. Chi-square test was used to determine risk factors for stillbirth in bivariate analysis. A multivariable regression model was used to estimate adjusted odds ratios (AOR) with 95% confidence intervals for maternal and fetal factors associated with stillbirth. A p-value of less than 0.05 was considered statistically significant.

**Results:**

The frequency of stillbirth was 3.5%. Pre-eclampsia (AOR 3.99; 95% CI: 3.31–4.81) and placental abruption (AOR 22.62; 95% CI: 15.41–33.19) were the strongest maternal risk factors associated with still birth. While non-cephalic presentation (AOR 6.05; 95% CI: 4.77–7.66) and low birth weight (AOR 9.66; 95%CI: 8.66–10.77) were the fetal factors with the greatest impact on stillbirth.

**Conclusion:**

The rate of stillbirth in our study was consistent with past studies of developing countries. Numerous maternal and fetal factors risk factors were identified. Early identification of at risk pregnancies and appropriate intervention may help to reduce the occurrence of stillbirth.

## Background

Stillbirth is defined as a baby born dead (without spontaneous respiration or heartbeat) with >1000 grams birth weight or ≥28 weeks gestation [1]. Stillbirths can be classified based on either physical appearance as macerated and fresh or time to death as death before labour and death occurs during labor [2–4]. Stillbirth has been associated with extensive psychosocial consequences for parents and family, and has been linked to post-traumatic stress disorder, anxiety and depression [1].

In 2015, the World Health Organization estimated that 2.6 million stillbirths occur each year globally, and ranged from 2·4–3·0 million [5]. These estimates correspond to 19% reduction in number of stillbirth since 2000. The vast majorities (98%) of stillbirths occur in low and middle-income countries, especially in sub Saharan Africa and south Asia (77%) [5]. Stillbirth rate is ten-fold higher in developing compared to developed countries [6–8]. Sub-Saharan Africa is particularly affected: 880,000 stillbirths occur annually, 60% of which affect poor and rural families [5,6]. In Tanzania, approximately 47,000 stillbirths occur each year. This corresponds to a rate of 25.9 per 1000 births: this is the ninth highest rate worldwide [9].

Numerous factors have been associated with an increased risk of stillbirth. Notable maternal factors include smoking, alcohol use, diabetes, multiparity ≥3, extreme maternal age, placental abruption, antepartum hemorrhage, and infection during pregnancy [5, 10–13]. Fetal factors include congenital malformation, birth asphyxia, growth restriction, and prolonged/obstructed labor [3, 5, 14]. Accordingly, certain interventions have been reported to reduce the incidence of stillbirth, such as antenatal ultrasound, infection management, and micronutrient supplementation [15] to mention a few. Unfortunately, few of these interventions are routinely used in low-income countries.

In Sub-Saharan Africa and Tanzania, few studies have explored the incidence and predictors of stillbirth. The majority of these studies are case-control studies or cross-sectional in nature which utilize the data on a small sample of pregnant women collected in a short period of time. The weakness of these kind of studies do not link the mother and baby outcomes due to limited follow-up time which make difficult to ascertain the exposure to outcome relationship [16–21].

Therefore, this study thus aimed to evaluate stillbirth risk factors using a more comprehensive database. This information would be useful to guide future preventative care efforts.

## Methods

### Study design and setting

This was a retrospective cohort study conducted using the birth registry of Kilimanjaro Christian Medical Centre (KCMC), a referral hospital located in Moshi, northern Tanzania. This hospital serves a catchment population of over 11 million, mostly drawn from the Kilimanjaro region and nearby communities. Averages of 4000 births occur at KCMC each year. The birth registry has been operational since 2000, during this period all deliveries that occurred at KCMC are recorded in a computerized database system at the medical birth registry. Medical birth registry it is located within hospital grounds at the Reproductive and Child Health Centre. A unique maternal hospital identification number was used to link records of subsequent deliveries of the same mother.

A stillbirth was defined as a baby born dead after 24 completed weeks of pregnancy and risk factors were defined as any condition, attribute, characteristic or exposure of the mother (for maternal risk factors) and fetus (fetal risk factors) that increases the likelihood of developing a stillbirth during pregnancy or delivery.

### Study population and sampling procedure

The study population was women delivered in KCMC hospital with singleton babies. All KCMC deliveries from January 2000 to December 2015 were considered for this study, a total of 50,177 births. Record with missing data on stillbirths was excluded. Pregnancies with multiple gestations were also excluded, to avoid over representation high-risk pregnancies. After such exclusion, 47,234 deliveries were analyzed.

### Data collection methods and tools

We conducted a historic cohort study using maternally linked data from Kilimanjaro Christian Medical Centre (KCMC) medical birth registry. A unique maternal hospital identification number was used to link records of subsequent deliveries of the same mother.

KCMC is one of the four zone referral hospitals in Tanzania, located in Moshi urban district, Kilimanjaro region in the Northern Tanzania. The centre receives deliveries from the nearby communities within the region and referred cases from other regions. The study population was women delivered in KCMC hospital with singleton babies. We excluded women with missing child status at birth, multiple births and neonatal death in labour ward in order to prevent overestimation of incidence of stillbirth.

**Fig 1:**
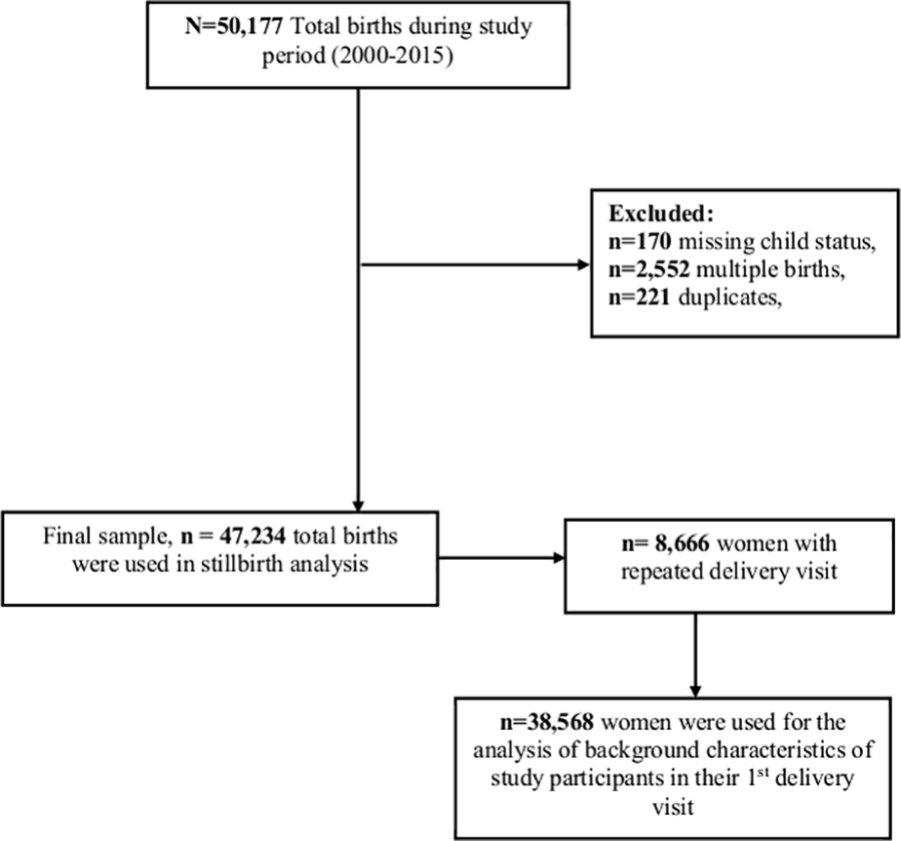
Schematic diagram for sample size estimation from Kilimanjaro Christian Medical Centre birth registry.

We constructed our cohort using 38,568 women who were recorded for the first pregnancy visit with a singleton delivery at KCMC in the period 2000–2015. Our final study sample was 47,234 births that were recorded with at least one or more birth during the follow-up period (Fig **1**). We studied maternal and fetal risk factors of stillbirth from the first recorded pregnancy to any of the subsequent pregnancies.

### Data collection method

We extracted data from KCMC medical registry which collects information from all women who deliver at the department of obstetrics and gynaecology in KCMC hospital. Data are collected within 24 hours after delivery or later on as mothers have recovered in case of complicated deliveries. Trained nurse midwives carried out daily interviews using a standardized questionnaire to collect the registry data. In addition, mothers admitted were asked to provide their antenatal (ANC) cards from which relevant data were abstracted. Data captured include parent’s socio-demographic characteristics, maternal health before and during present pregnancy, complications during labour and delivery, and information from the interview regarding the mother’s previous pregnancies including miscarriage, stillbirth, preterm birth and fertility treatment. Also Women were asked regarding present and past cigarette, alcohol, and chewing tobacco use of family planning methods.

Furthermore, information on the baby such as sex, date and time of delivery, birth weight, gestational age, presentation, length and head circumference, plurality, mode of delivery, abnormal conditions (birth defects, injuries or other diseases), Apgar score, and child status were recorded.

The main outcome variable was child status which was recorded into four categories; 1) live born 2) live born transferred to NCU 3) neonatal death in labour ward 4) stillborn and further categorized into either stillborn or live birth by excluding neonatal death in labour ward. Stillborn in our study was defined as baby born dead (without spontaneous respiration or heartbeat) with >1000 grams birth weight or ≥28 weeks gestation [1].

### Data analysis

Data analysis was performed using Statistical Package for the Social Sciences (SPSS) version 20.0. Mean and SD was used to describe continuous variable and comparison of proportions was performed by Pearson chi-square (χ2) for categorical variables to determine associations between demographic variables and the outcome of interest. Multivariable Log-binomial regression model was used to estimate the odds ratios (ORs) for stillbirth with 95% confidence intervals (CIs). A p-value of less than 5% was considered statistically significant. We used mothers as the primary unit for our analysis and conducted a clustered analysis technique with robust estimation of variances to account for the correlation between repeated observations of the same woman. Odds ratio was used to measure the strength of association instead of risk ratio due to rarity of stillbirth.

### Ethical considerations

Ethical approval was sought from Kilimanjaro Christian Medical University college ethics committee. For practical reasons, the midwives give oral information individually to each mother and then asked if is willing to participate. Participation and further interviewing was then based on oral consent. Following consent, the mother could still choose not to reply to single question. In order to document the consent process, the birth registry has an instruction manual for registration of deliveries in the registry. This manual contains the standard operating procedures on how to conduct the interview and also has a description of the basic rules including ethical guidelines such as consent process. The consent procedure was approved by the ethics committee as it was part of the study protocol.

## Results

### Socio-demographic characteristics of the study participants

Table **1** show the socio-demographic characteristics of the study participants. A total of 38568 singleton deliveries recorded for the first time were analyzed. Of these, 37096 (96.2%) were live births and 1472 (3.8%) stillbirths. There were 1472 mothers who delivered a stillborn fetus at 28 weeks or later at KCMC Hospital and 187 had recurrence of stillborn during the study period this resulted in a total of 1659 stillbirths’ fetuses (Table 1). Of these stillborn fetuses, 623 (37.6%) were recorded as fresh stillborn, 586 (35.3%) macerated stillborn and 450 (27.1%) had missing stillborn information based on physical appearance. As noted, the analysis was restricted to all 1659 fetuses with adequate data for analysis. The maternal demographics of this subgroup were not substantially different from those of the full group of mothers in the dataset (Table 1).

**Table 1:** Socio-demographic characteristics of the study participants at first recorded pregnancy (N = 38568).

Characteristics	Total n (%)	Still birth n (%)	Live birth n (%)	χ^2^ P-value
**Age (years) †**	**26.9(6.04)**	28.5 (6.47)	26.8 (6.01)	
<25	17420 (45.2)	545 (3.1)	16869 (96.9)	<0.001
25–34	17385 (45.1)	689 (4.0)	16692 (96.0)	
≥35	3763 (9.8)	238 (6.3)	3535 (93.7)	
Residence*				
Rural	16961 (44.0)	860 (5.1)	16115 (94.9)	<0.001
Urban	21516 (55.8)	609 (2.8)	20894 (97.2)	
Missing	89 (0.2)	3 (3.3)	87 (96.7)	
Education*				
Illiterate	806 (2.1)	63 (7.8)	743 (92.2)	<0.001
Primary	22105 (57.3)	990 (4.5)	21115 (95.5)	
Secondary	4080 (10.6)	125 (3.1)	3956 (96.9)	
Higher	11577 (30.0)	294 (2.5)	11282 (97.5)	
Marital status*				
Married	32863 (85.2)	1258 (3.8)	31611 (96.2)	0.892
Single	5666 (14.7)	213 (3.8)	5446 (96.2)	
Missing	40 (0.1)	1 (2.5)	39 (97.5)	
Occupation*				
Employed	12725 (33.0)	372 (2.9)	12354 (97.1)	<0.001
Unemployed	2568 (66.6)	1098 (4.3)	24584 (95.7)	
Missing	159 (0.4)	2 (1.3)	158 (98.7)	

†Mean (SD)

*Missing values; SD-Standard Deviation

The mean (±SD) age of the participants was 26.9 (6.04) years with the majority of women were below 35 years (90.2%). More than half (55.8%) of participants resided in an urban area, majority (57.30%) had primary education and Majority of the women (66.6%) were employed. Furthermore, Women with stillbirth in their first recorded pregnancy were older (P<0.001), residing in rural areas (P<0.001), illiterate (P<0.001) and unemployed (P<0.001) compared with women with live birth.

### Maternal factors associated with stillbirth

Table **2** displays the associations between maternal factors and stillbirth. In crude analysis, maternal factors associated with stillbirth were: advanced maternal age, pregnancy induced hypertension (PIH), number of antenatal care (ANC) visits, alcohol consumption during pregnancy, gestational diabetes mellitus, pre-eclampsia, and anemia. HIV status and eclampsia had no significant association with stillbirth. After adjusting for the confounders, the following factors remained significantly associated with stillbirth. These include maternal age ≥35years (AOR 1.51, 95% CI: 1.26–1.81), alcohol use during the index pregnancy (AOR 1.37, 95% CI: 1.17–1.61), pre-eclampsia (AOR 4.10, 95% CI: 3.38–4.97), and placental abruption (AOR 35.55, 95% CI: 22.77–55.50), Anaemia (AOR 1.69, 95% CI: 1.17–2.44), and antenatal care visits less than 4 (AOR 2.12, 95% CI: 1.83–2.45). On the other hands, premature rupture of membrane (PROM) (AOR 0.32, 95% CI: 0.15–0.68) offered protection against stillbirth.

**Table 2:** Clustered logistic regression to determine maternal factors associated with still birth in Northern Tanzania (N = 47,234).

Characteristic	Total	Still Birth (n = 1659)	COR [95% CI]	AOR [95% CI]
**Age group (years)**				
<25	19257	581 (3.0)	0.85[0.76–0.95]	0.96[0.84–1.11]
25–34	22778	805 (3.5)	1.0	1.0
≥35	5199	273 (5.25)	1.51[1.31–1.75]	1.51[1.26–1.81]
**HIV status**				
Negative	33467	1045 (3.1)	1.0	1.0
Positive	1913	72(3.8)	1.21[0.95–1.55]	1.23[0.95–1.60]
**PIH**				
No	47123	1647 (3.5)	1.0	1.0
Yes	111	12 (10.8)	3.01[1.98–4.58]	2.78[0.26–29.16]
**Number of ANC visit**				
> 4	19086	398 (2.1)	1.0	1.0
< 4	27299	1180 (4.3)	2.12[1.89–2.38]	2.12[1.83–2.45]
**Alcohol**				
No	33143	1222 (3.7)	1.0	1.0
Yes	13870	423 (3.1)	1.22[1.09–1.36]	1.37[1.17–1.61]
**Gestational Diabetes**				
No	47105	1648 (3.5)	1.0	1.0
Yes	129	11 (8.5)	2.57[1.31–5.03]	0.83[0.09–7.19]
**Pre-eclampsia**				
No	45433	1437 (3.2)	1.0	1.0
Yes	1801	222 (12.3)	4.30[3.71–4.99]	4.10[3.38–4.97]
**Eclampsia**				
No	47171	1665 (3.5)	1.0	1.0
Yes	63	4 (6.4)	1.86[0.68–5.14]	1.87[0.44–7.93]
**Anemia**				
No	46469	1615 (3.5)	1.0	1.0
Yes	765	44 (5.8)	1.61[1.20–2.16]	1.69[1.17–2.44]
**Abruption of placenta**				
No	47077	1569 (3.3)	1.0	1.0
Yes	157	90 (57.3)	38.96[28.34–53.56]	35.55[22.77–55.50]
**Placenta Previa**				
No	47129	1652 (3.5)	1.0	
Yes	105	7 (6.7)	1.97[0.91–4.24]	
**PROM**				
No	46206	1644 (3.6)	1.0	1.0
Yes	1028	15 (1.5)	0.40[0.24–0.67]	0.32[0.15–0.68]

Adjusted OR: Adjusted for maternal age, PIH, number of ANC visits alcohol consumption during pregnancy, GDM, abruption of placenta pre-eclampsia, PROM and anemia.

### Fetal factors associated with stillbirth

Numerous factors were associated with stillbirth in crude analysis (Table **3**). After adjusting for confounders non-cephalic fetal presentation (AOR 5.64, 95% CI: 4.42–7.19), pre-term birth (AOR 1.43, 95% CI: 1.22–1.68), Post-term (AOR, 1.55 95% CI: 1.02–2.35), birth weight >4000Kg (AOR 2.34, 95%CI: 1.83–2.97), birth weight < 2500 (AOR 9.98, 95%CI: 8.95–11.13) were significantly associated with an increased odds of stillbirth. Non-spontaneous delivery and other modes of delivery were found to be protective against stillbirth (AOR 0.73, 95% CI: 0.63–0.82), (AOR, 0.81, 95%CI: 0.73–0.91) respectively.

**Table 3:** Clustered logistic regression to determine fetal factors associated with still birth in Northern Tanzania (N = 47,234).

Characteristics	Total	Still Birth (n = 1659)	COR [95% CI]	AOR [95% CI]
**Fetal presentation**				
Cephalic	46477	1525 (3.3)	1.0	1.0
Non-cephalic	650	127 (19.5)	7.16[5.86–8.75]	5.64[4.42–7.19]
**Gestational age (weeks)**				
Term (37–42)	9207	186 (2.0)	1.0	1.0
Pre-term (<37)	37154	1444 (3.9)	1.96[1.68–2.29]	1.43[1.22–1.68]
Post term (>42)	873	29 (3.3)	1.67[1.12–2.48]	1.55[1.02–2.35]
**Birth weight(grams)**				
2500–4000	40391	748 (1.9)	1.0	1.0
<2500	4943	828 (16.8)	10.66[9.60–11.48]	9.98[8.95–11.13]
>4000	1900	83 (4.4)	2.42[1.92–3.06]	2.34[1.83–2.97]
**Malformation**				
No	47167	1655 (3.5)	1.0	1.0
Yes	67	4 (5.9)	1.75[0.64–4.80]	0.85[0.23–3.12]
**Mode of delivery**				
SVD	30892	1036 (3.4)	1.0	1.0
Others	16227	601 (3.7)	1.11[1.-1.23]	0.81[0.73–0.91]

Adjusted OR: Adjusted for Fetal presentation, Gestational age, Birth weight, Malformation, Abruption of placenta, Placenta Previa, PROM, Mode of delivery.

## Discussion

In this study, the proportion of stillbirth was 3.8%.There was a slight difference in stillbirth rate between fresh and macerated stillbirth (1.3% vs. 1.2%, respectively). About one percent was unspecified stillbirths. Independent maternal risk factors for stillbirth were: maternal age >35 years, lower ANC visits alcohol use during pregnancy, pre-eclampsia, anaemia, PROM and placental abruption. Independent fetal risk factors for stillbirth were: non-cephalic presentation, birth weight <2500 grams, birth weight >4000 grams, and pre-term birth and other modes of delivery.

The proportion of stillbirth in this study was notably higher than the proportion of 0.42% reported in the U.K [11]. However, it was comparable with 3.26% that was reported by Kidanto and colleagues in Tanzania [22], 2.59% by Chalumeau and colleagues in urban West Africa [23], and and 4.0% by Avachat and colleagues in India [14]. These differences in proportions are likely attributable to population health differences between developed and developing countries, as well as the availability of prenatal and obstetric emergency care.

We found maternal age ≥35 years and obesity to be associated with increased odds of stillbirth. These risk factors were also found in two UK studies [12, 13]. Both maternal age and obesity have cumulative negative effects on vascular health, which may have negative impacts on the developing fetus. Placental abruption connoted a remarkable thirty five-fold increase in risk of stillbirth, similar to Kidanto et al. [22]. However, our study found that PROM had a protective effect against stillbirth, contrary to the findings of Kidanto. Our study took place at a large medical center: it is possible that women with PROM were more likely to be referred to KCMC, where they received aggressive obstetric care to prevent stillbirth.

We found pre-eclampsia and alcohol use during pregnancy to be statistically significant risk factors for stillbirth. This has not been commonly reported in previous studies [14, 23, 24]. However, the association is physiologically plausible: alcohol is a known toxin to the fetus, and disturbed placental function plus impaired remodeling of the spiral artery in pre-eclampsia could lead to low birth weight, a known risk factor for stillbirth.

The fetal factors of birth weight <2500 grams and prematurity (gestational age<37 weeks) had significant positive associations with stillbirth, consistent with studies of rural India and Tanzania [14, 23]. Infants with prematurity and/or low birth weight may have a high risk of death due to their underdeveloped respiratory system. Non-cephalic presentation and high birth weight (>4000 grams) were also associated with stillbirth. This association was not reported in previous studies conducted by Chalumeau, Kidanto, and Gardosi [23–24], although similar findings of relationship between birth weight and stillbirth was reported by studies conducted in Iran, Zambia and Ghana [25–27]. However, the increased risk may be explained by difficulty in delivery of a large or malpresented infant, leading to prolonged labor, fetal distress, and death. Our study did not find an association between congenital anomaly and stillbirth.

### Strengths and limitations of the study

This study utilized a large sample of women of reproductive age. This provided us with high power to detect the association between exposure variables and outcome of interest. Most of the associations found were consistent with the results of prior studies, providing further confidence in our analysis. However, some limitations need to be taken into account when interpreting our results.

First, this study used hospital-based data which had already been recorded to perform different research projects and not specifically meant for this study. We had no control for some variables which were missing despite that they were important in the present study.

Second, this study took place at a referral and teaching hospital. It is possible that KCMC sees a disproportionate number of complex deliveries, leading this study to overestimate the proportion of stillbirth. Due to higher proportion of home delivery rate (36%) in Tanzania the reported still birth rate in our study may be underestimated, however home delivery rate in the study setting is approximately 8% thus the reported still birth rate may reflect the true still rate in the studied population [28].

Finally, some of birth registry entries were incomplete especially on time to death, specification of both fresh or macerated stillbirth and mothers smoking status as well as weight or height in both prepregnancy and on admission. Although smoking and prepregnancy BMI was not assessed in this study due to low prevalence of smoking among Tanzanian women and missing data on prepregnancy weight, future studies could, depending on cultural context and common practice, include data collection on smoking habits and prepregnancy weight.

### Conclusion

The global stillbirth epidemic has reached women in developing countries, our study found the higher incidence of stillbirth than typically found in the developed countries. Maternal socio-demographic and obstetric characteristics are associated with increased risks of stillbirth. Health care professionals should identify increased risk of stillbirth with every woman of childbearing age in order to address in a timely manner the preventable and modifiable risk factors of stillbirth. Access of these women to targeted counselling and prevention programs may assist in improving the wellbeing of these women. It is important to pay more attention to maternal influences before pregnancy to prevent the recurrence cycle of stillbirth. Strategies to raise public awareness of the risks of stillbirth on mothers and offspring’s health are required.
